# Transcriptomic and Proteomic Profiling of Rabbit Kidney Cells Infected with Equine Herpesvirus 8

**DOI:** 10.3390/v17050647

**Published:** 2025-04-29

**Authors:** Yanfei Ji, Dandan Xu, Wenxuan Si, Yu Zhang, Muhammad Zahoor Khan, Xia Zhao, Wenqiang Liu

**Affiliations:** Department of Veterinary Medicine, School of Agriculture and Biology, Liaocheng University, Liaocheng 252000, Chinazahoorkhan@lcu.edu.cn (M.Z.K.)

**Keywords:** EHV-8, RK-13 cells, proteomics, transcriptomics, immune response, TNF signaling pathway

## Abstract

The present study investigated the host cell response to EHV-8 infection in rabbit kidney (RK-13) cells through transcriptomic and proteomic approaches. At 24 h post-infection, a total of 2118 differentially expressed genes (DEGs) were identified, with 1338 upregulated and 780 downregulated. At 48 h, 7388 DEGs were detected, with 4342 upregulated and 3046 downregulated genes. Proteomic analysis revealed 932 differentially expressed proteins (DEPs) at 24 h (364 upregulated and 568 downregulated) and 3866 DEPs at 48 h (2285 upregulated and 1581 downregulated). Of these, 237 upregulated and 336 downregulated proteins were common across both time points. Kyoto Encyclopedia of Genes and Genomes (KEGG) pathway analysis indicated that the majority of DEGs and DEPs were enriched in key inflammation-related pathways, notably the TNF and NF-κB signaling pathways. Validation of the transcriptomic and proteomic data was performed using RT-PCR and parallel reaction monitoring (PRM), respectively, and confirmed consistent trends for TNFR1, NF-κB p65, and MAP3K8, as reported in the transcriptomic and proteomic screening. These findings suggest that EHV-8 infection may modulate host immune responses by activating the TNF signaling pathway. However, given that RK-13 cells may not fully replicate viral–host interactions in equine species, further in vivo studies in horses and donkeys are required to provide a more comprehensive understanding of the viral pathogenesis in these animals.

## Simple Summary

Equine herpesvirus type 8 (EHV-8), a member of the alphaherpesvirus subfamily, is a significant pathogen in donkeys and causes abortion and respiratory infections, which result in considerable economic losses within the equine industry. Despite its importance, limited research has been conducted on the molecular response to EHV-8 in donkeys. The present study investigated the host cell response to EHV-8 infection in rabbit kidney (RK-13) cells through transcriptomic and proteomic approaches. Our findings identified several candidate genes and proteins, along with their associated signaling pathways, involved in the cellular response to EHV-8 infection in this in vitro model. However, because RK-13 cells may not accurately replicate viral–host interactions in equine species, additional in vivo studies in horses and donkeys are necessary to achieve a more thorough understanding of viral pathogenesis in these animals.

## 1. Introduction

Equine herpesviruses (EHVs) represent a significant group of viral pathogens affecting equid species and cause respiratory disease; reproductive failure, including abortion; and neurological disorders with potentially fatal outcomes [1]. Currently, nine distinct EHV subtypes (EHV-1 through EHV-9) have been characterized. Among these, EHV-1 and EHV-4 are the predominant pathogens, with horses as their natural reservoir hosts. These viruses are etiological agents of equine rhinopneumonitis, abortion in pregnant mares, and neonatal mortality, and they constitute a substantial economic burden to the global equine industry [2,3,4]. EHV-8, classified within the alphaherpesvirus subfamily alongside EHV-1 and EHV-4, has been associated with respiratory pathology and abortion in donkeys [5]. In China, a leading country in donkey production, the industry has undergone a transition from service-oriented to commercial applications, resulting in a greater research focus on EHV-8 compared to EHV-1/EHV-4 [6]. Despite this increased attention, EHV-8 remains understudied, with significant knowledge gaps regarding its pathogenesis and host–pathogen interactions.

Contemporary advances in high-throughput transcriptomic and proteomic technologies provide sophisticated platforms for the comprehensive analysis of gene expression profiles and protein abundance patterns associated with biological processes and molecular pathways. These omics methodologies are particularly valuable for elucidating pathogenic mechanisms in infectious disease contexts. However, single-omics approaches often provide limited perspective on complex disease mechanisms. The integration of transcriptomic and proteomic datasets facilitates a more comprehensive understanding of biological systems, as it captures the dynamic interplay between gene expression and protein function [7]. Consistent with this integrated approach, previous investigations employing transcriptomic and proteomic analyses have demonstrated the upregulation of innate immunity-related genes in caprine herpesvirus 1-infected Madin-Darby bovine kidney cells [8]. Additionally, RNA sequencing of EHV-1-infected peripheral blood mononuclear cells has revealed the dysregulation of immune and coagulation pathways, with interleukin-6 (IL-6) upregulation, aberrant T-cell activation, and altered progesterone signaling observed in equine myeloencephalopathy cases [9]. To date, no comprehensive transcriptomic or proteomic characterization of host cellular responses to EHV-8 infection has been reported in the literature. The present study constitutes the first investigation of the effects of the EHV-8 strain on Rabbit Kidney 13 (RK-13) cells at both transcriptomic and proteomic levels. These findings provide preliminary insights into EHV-8 pathogenesis, establishing a foundation for subsequent investigations into virus–host interactions and immune mechanisms associated with this pathogen.

## 2. Materials and Methods

### 2.1. Test Material

RK-13 cells were purchased from Wuhan Punosai Life Technology Co., Ltd., China. The cells were cultured in Modified Eagle Media (MEM, Dalian Meilun Biotechnology Co., Ltd., Dalian, China) medium, supplemented with 10% fetal bovine serum (FBS, Dalian Meilun Biotechnology Co., Ltd., Dalian, China) at 37 °C with 5% CO_2_. Cells were inoculated into cell culture flasks, and viruses were inoculated in a monolayer stage. EHV-8 LCDC01 was isolated from nasal swabs of donkeys from a large-scale donkey farm in Liaocheng, Shandong, China (GenBank: PRJNA787358). A total of 200 µL (TCID50 = 10^−3.75^/100 μL) viral suspension was added to 800 µL modified MEM medium. The cells were cultured at 37 °C for 1 h, and the medium was then discarded. The cells were washed with phosphate buffer salt solution (PBS), and 5 mL of fresh and complete culture solution was added to the culture bottle. For the control group, cells were inoculated with 200 µL of sterile PBS instead of virus.

### 2.2. RNA Extraction

Total RNA was extracted from RK-13 cells at 24 h and 48 h post-infection using Trizol reagent (Beijing Tianjingsha Gene Technology Co., Ltd., Beijing, China). Cells were briefly mechanically lysed and homogenized in Trizol reagent. The homogenate was transferred to RNase-free microcentrifuge tubes and incubated for phase separation. Subsequently, 200 μL of cold chloroform (Shanghai Sinopharm Group Chemical Reagent Co., Ltd., Shanghai, China) was added, and the mixture was vortexed thoroughly. After 10 min incubation at room temperature, the samples were centrifuged at 12,000× *g* for 15 min at 4 °C. The resulting aqueous phase was carefully transferred to fresh tubes containing 500 μL of cold isopropanol (Shanghai Sinopharm Group Chemical Reagent Co., Ltd.). Following 10 min of incubation, the samples were centrifuged at 12,000× *g* for 10 min at 4 °C to precipitate RNA. The supernatant was carefully aspirated, and the RNA pellet was retained. Genomic DNA contamination was eliminated using Takara DNA-OFF^®^ (Baojirushi Physical Technology Co., Beijing, China). The purified RNA samples were transferred to RNase-free cryovials, flash-frozen in liquid nitrogen for 30 min, and stored at −80 °C until further analysis.

### 2.3. Protein Extraction

Cell cultures were prepared following the previously described protocol (Section 2.1). Cells were harvested by suspension in PBS and transferred to RNase-free tubes at a standardized concentration of 1 × 10^7^ cells/mL (verified by cell counting). For lysis, 50 μL of RIPA buffer (Radio Immunoprecipitation Assay, Shanghai Biyuntian Biotechnology Co., Ltd.) supplemented with 1 mM PMSF (Phenylmethanesulfonyl fluoride, Shanghai Biyuntian Biotechnology Co., Ltd., Shanghai, China) and 2% phosphatase inhibitor cocktail (Wuhan Xavier Biotechnology Co., Ltd., Wuhan, China) was added to each sample. The cell suspensions were incubated with continuous agitation for 20 min to ensure complete lysis. Following incubation, the lysates were centrifuged at 13,000× *g* for 30 min at 4 °C. The protein-containing supernatants were carefully collected and quantified using the Bicinchoninic Acid Assay (BCA) Protein Assay Kit (Shanghai Biyuntian Biotechnology Co., Ltd.), according to the manufacturer’s instructions. The quantified protein samples were transferred to cryogenic storage tubes, flash-frozen in liquid nitrogen for 30 min, and stored at −80 °C until further analysis.

### 2.4. Transcriptome Sequencing

The collected cell samples were subjected to RNA-seq transcriptome sequencing by Meiji Biotechnology Co., Ltd. Transcriptome sequencing included total RNA extraction, Oligo dT enrichment of mRNA, the fragmentation of mRNA, the reverse synthesis of cDNA, the linking of the adapter, and computer-based sequencing on the Illumina platform. The gene expression levels of each sample were analyzed using Kallisto v0.46.1 (https://pachterlab.github.io/kallisto/download (accessed on 24 August 2022)) software, and the results were analyzed using DESeq2 (https://bioconductor.org/packages/stats/bioc/DESeq2/ accessed on 13 April 2023) software for differential gene expression levels. Based on the Fragments per Kilobase of Exon per Million Fragments Mapped (FPKM) method, significantly differential expressed genes were determined using the following criteria: log2FC (fold change) > 1 and adjusted *p* < 0.05. The screened differentially expressed genes (DEGs) were imported into the KEGG database to determine their regulated biological functions and pathways.

### 2.5. Proteome Sequencing

The protein (100 μg) was combined with Triethylammonium bicarbonate (TEAB) (Shanghai Sinopharm Chemical Reagent Co., Ltd., Shanghai, China) and Tris 2-carboxyethyl phosphine hydrochloride (TCEP) (Shanghai Sinopharm Chemical Reagent Co., Ltd.) at a final concentration of 10 mM for 60 min at 37 °C. Iodoacetamide (IAM) (Shanghai Sinopharm Chemical Reagent Co., Ltd.) was then added at a final concentration of 40 mM and allowed to react for 40 min at room temperature in the dark. The precipitate was sufficiently dissolved with TEAB. This was followed by the addition of trypsin at a mass ratio of 1:50 (enzyme) and overnight digestion at 37 °C. Acetonitrile (Shanghai Sinopharm Chemical Reagent Co., Ltd.) was added to the TMT reagent (Shanghai Biyuntian Biotechnology Co., Ltd.), vortexed, and centrifuged, and one tube of the reagent mixture was added for every 100 μg of peptide and incubated at room temperature for 2 h. Hydroxylamine (Shanghai Sinopharm Chemical Reagent Co., Ltd.) was added and allowed to react for 30 min at room temperature. Equal amounts of labeled products were mixed and evacuated using a vacuum concentrator. Liquid chromatography tandem mass spectrometry (Evosep One coupled with Orbitrap Exploris 480 mass spectrometer, Shanghai Meiji Bio-medical Technology Co., Ltd., Shanghai, China) was used for the analysis. Peptides were dissolved in mass spectrometry upload buffer, added to the sample, and separated on a column for 44 min at a flow rate of 300 nL/min. The MS scanning range (m/z) was 350–1500, the acquisition mode was DDA, and the fragmentation mode was HCD; the resolution of the primary mass spectrum was 60,000, and the resolution of the secondary mass spectrum was 15,000, with a dynamic exclusion time of 30 s. The intelligent acquisition of Turbo TMT improved the resolution of reported ion isotopes.

Proteome Discoverer™ Software 2.4 (Shanghai Meiji Bio-medical Technology Co., Ltd.) was used for the analysis, and the database used for the search was the NCBI Rabbit Database (https://www.ncbi.nlm.nih.gov/, accessed on 24 August 2022). Precursor Mass Tolerance was set at 20 ppm, and Fragment Mass Tolerance was set at 0.02 Da. The false discovery rate (FDR) of peptide identification was set as FDR ≤ 0.01. A minimum of one unique peptide identification was used to support protein identification. Similar to the transcriptome data analysis, the proteomics data were analyzed by evaluating the ratio of the relative protein expression in the different groups and the *p*-value of statistical tests for differences. The significance of differentially expressed proteins was calculated using criteria log2 FC > 1.2 and adjusted *p* < 0.05. The KEGG pathway enrichment analysis for the differentially expressed proteins was conducted to determine the main pathways in which DEPs were involved.

### 2.6. Integrative Analysis of Transcriptome and Proteome

To investigate the key factors and critical molecular mechanisms associated with the EHV-8 infection of RK-13 cells, a transcriptome-proteome nine-quadrant association analysis was performed via the Majorbio Cloud platform (cloud.majorbio.com), and quadrants where both genes and proteins were upregulated (rho > 0) were selected.

### 2.7. Functional Enrichment Analysis

Functional analysis was performed for differentially expressed transcripts by subjecting them to KEGG (with *p* < 0.05 as the criterion for significant expression (https://www.kegg.jp/kegg, accessed on 24 August 2022)).

### 2.8. Quantitative Reverse Transcription PCR (RT-qPCR) Validation for RNA-Seq Analysis

To confirm the RNA-Seq results, RT-qPCR was performed on control and 48 h infected groups, total RNA from three technical replicates was extracted, and the expression levels of the four genes were examined using the PrimeScript™ FAST RT kit and the gDNA Eraser kit (Baojirushi Physical Technology Co., Ltd., Beijing, China), according to the manufacturer’s protocol. Primers were designed online and synthesized using Shanghai Bioengineering Primer Designer Software 5.0 (https://store.sangon.com/newPrimerDesign, accessed on 24 August 2022), and the primers were used for the detection of the expression levels of the four genes using TB Green^®^ Premix Ex Taq™ II (Tli RNaseH Plus) (Baojirushi Physical Technology Co., Beijing, China) for RT-qPCR analysis. Using a 25 μL reaction volume, 8.5 μL ddH2O, 1 μL forward primer, 1 μL reverse primer, 12.5 μL 1× TB Green^®^ Premix Ex Taq™ II, and 2 μL cDNA template were added. The qPCR assay was performed using the CFX96 Touch Real-Time PCR Detection System (Bio-Rad Laboratories, Hercules, CA, USA). GAPDH was used as the reference gene, and the gene expression level was calculated by the 2^−ΔΔCt^ method [10]. Information about the primers is provided in Annex S1 of the Supplementary File.

### 2.9. Parallel Reaction Monitoring (PRM) Targeting Protein

In this study, protein identification, which was based on the TMT results, was performed using Proteome Discoverer 2.5 software. Subsequently, Parallel Reaction Monitoring (PRM) technology was employed for targeted quantitative analysis of the relevant proteins. The analysis was conducted using a nanoElute2 liquid chromatography system coupled to a timsTOF Pro time-of-flight mass spectrometer (Bruker, Billerica, MA, USA) with an IonOpticks Aurora integrated column (25 cm × 75 µm, IonOpticks, Fitzroy, Australia). The chromatographic flow rate was maintained at 200 nL/min. Mobile phase A consisted of 0.1% formic acid aqueous solution (Sinopharm, Beijing, China), while mobile phase B was 0.1% formic acid in acetonitrile (Sinopharm, Beijing, China). The chromatographic gradient was programmed as follows: 0 min, 2% B; 45 min, 22% B; 50 min, 37% B; 55 min, 80% B; and 60 min, 80% B. Data processing was performed using Skyline software version 24.1 with a cut-off value of 0.95. For each peptide, the six fragment ions with the highest signal intensity were selected for analysis, and the fragment ion exhibiting the highest signal intensity chosen for quantification.

### 2.10. Western Blot

Sodium dodecyl sulfate polyacrylamide gel electrophoresis (SDS-PAGE) was performed by loading equal amounts of total protein onto a discontinuous gel consisting of a 5% stacking gel and a 12% resolving gel. Electrophoresis was conducted at 120 V for 30 min. The proteins were transferred to polyvinylidene difluoride (PVDF) membranes (Shanghai Biyuntian Biotechnology Co., Ltd.) in an ice bath using the sandwich method (100 V, 1 h). Following transfer, each membrane was washed twice with Tris-buffered saline containing 0.1% Tween-20 (TBST; Shanghai Biyuntian Biotechnology Co., Ltd.) and blocked with 5% non-fat dry milk in TBST (Shanghai Biyuntian Biotechnology Co., Ltd.) for 1 h at room temperature. The membrane was washed three times with TBST to remove excess blocking solution, then incubated with primary antibody overnight at 4 °C. Following incubation, the membrane was washed three times with TBST and subsequently incubated with horseradish peroxidase (HRP)-conjugated Goat Anti-Rabbit IgG H&L (1:10,000; Abclonal Trading Co., Ltd., Shanghai, China) for 1 h at room temperature. After incubation, the membrane was washed five times with TBST to remove unbound secondary antibody. Protein bands were visualized using Enhanced Chemiluminescence (ECL) substrate (Shanghai Biyuntian Biotechnology Co., Ltd., Shanghai) and documented using a chemiluminescence imaging system. Band intensities were quantified using ImageJ 1.4.3.67 software. Detailed information regarding the antibodies used for Western blotting is provided in Supplementary Material Annex S1.

## 3. Results

### 3.1. Analysis of Transcriptome and Proteome Data

**Figure 1:**
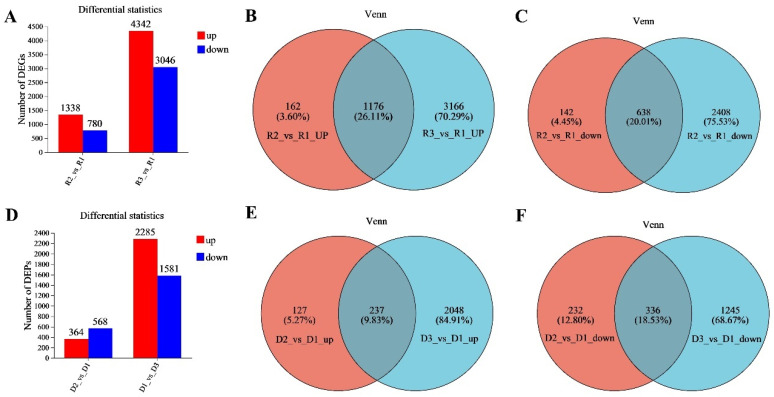
Differential expression analysis of the transcriptome and proteome in the two infection groups. (**A**) Differentially expressed genes (DEGs) between the two infection groups as compared to the control group. (**B**) Upregulated and downregulated DEGs shared by the two infection groups. (**C**) Downregulated DEPs common to both infection groups. (**D**) Differentially expressed proteins (DEPs) between the two infection groups as compared to the control group. (**E**) The two infection groups jointly upregulate proteomic DEPs. (**F**): The two infection groups jointly downregulate proteomic DEPs. Note: R1 represents the control group, R2 represents the 24 h infection group, and R3 represents the 48 h infection group. D1 represents the control group, D2 represents the 24 h infection group, and D3 represents the 48 h infection group. Note: For the comparison of different groups, *p*-values are those derived from the *t*-test.

Transcriptomic and proteomic analyses were performed on the EHV-8-infected RK-13 cell samples to evaluate gene and protein expression changes in response to infection. As illustrated in Figure 1A, the transcriptome of the 24 h infection group exhibited significant changes compared to the control group, with a total of 2118 DEGs identified. Among these, 1338 genes were upregulated, and 780 genes were downregulated (Supplementary Table S1). In contrast, the 48 h infection group displayed a larger set of DEGs, with 7388 genes showing differential expression compared to the control. Of these, 4342 genes were upregulated, while 3046 genes were downregulated (Supplementary Table S2). Venn diagram analysis (Figure 1B,C) was used to compare the DEGs between the two infection time points. The analysis revealed that 1824 DEGs were shared between the 24 h and 48 h infection groups. Of these, 1176 were upregulated, and 638 were downregulated relative to the control group (Supplementary Table S3).

Furthermore, proteomic profiling (Figure 1D) also revealed substantial changes in protein expression following infection. In the 24 h infection group, 364 proteins were upregulated, and 568 proteins were downregulated compared to the control (Supplementary Table S4). In the 48 h infection group, a larger set of DEPs was identified, with 2285 proteins upregulated and 1581 proteins downregulated (Supplementary Table S5). Venn diagram analysis of the proteomic data (Figure 1E,F) showed that 237 upregulated DEPs and 336 downregulated DEPs were common to both the 24 h and 48 h infection groups (Supplementary Table S6).

### 3.2. KEGG Pathway Enrichment Analysis of the Transcriptome

**Figure 2:**
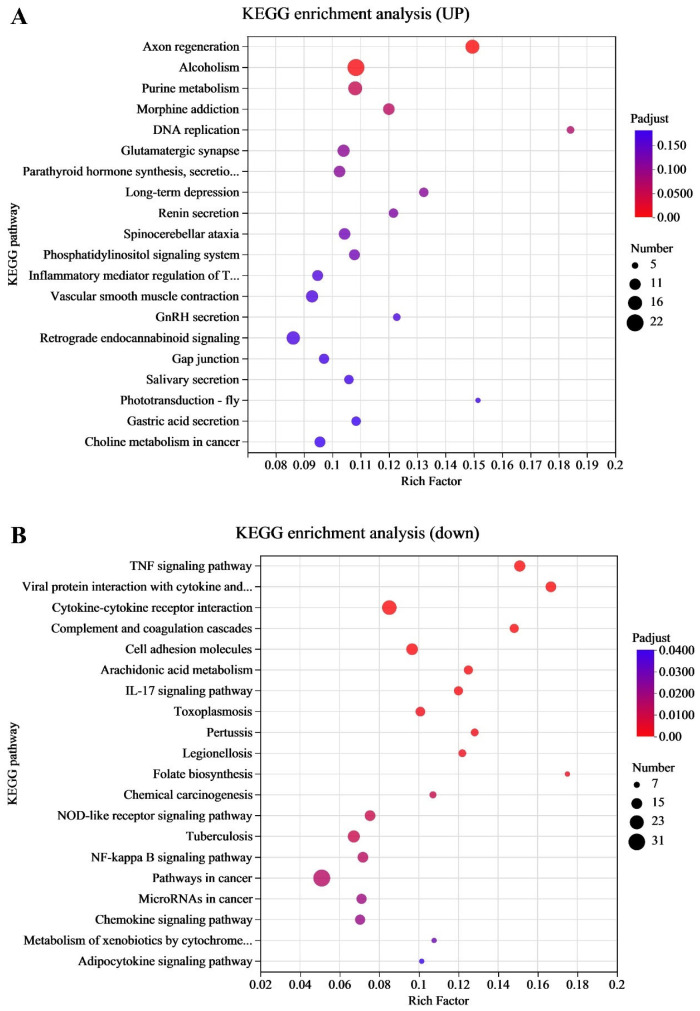
Transcriptome KEGG pathways enrichment analysis. (**A**) Graph of the common upregulation KEGG enrichment analysis in the transcriptome for the 24 h and 48 h groups. (**B**) Graph of the common downregulation KEGG enrichment analysis in the transcriptome for the 24 h and 48 h groups. Note: for the comparison of different groups, *p*-values are those derived from the *t*-test.

To identify the associated functions of DEGs involved in immune and metabolic pathways during EHV-8 infection, we performed KEGG pathway enrichment analysis. The data from the 24 h and 48 h infection groups were mapped to the KEGG database, and enrichment analysis was conducted for biological processes, molecular functions, and cellular components. As illustrated in Figure 2A, the top 20 pathways enriched for upregulated DEGs included key signaling pathways such as the tumor necrosis factor (TNF) signaling pathway, the NF-κB signaling pathway, the regulation of synaptic transmission, DNA replication, and cell adhesion through cell membrane adhesion molecules. These findings suggest that the involvement of immune response and cellular communication pathways in the host’s response to EHV-8 infection is significant. Figure 2B, on the other hand, highlights the pathways enriched for downregulated DEGs, which include the chemokine signaling pathway, exosome release, viral protein interactions with cytokines and cytokine receptors, and the complement and coagulation cascades. These results suggest a potential suppression of immune response mechanisms and alterations in extracellular vesicle-mediated signaling during the course of infection. The DEGs and their related pathways are provided in Supplementary Tables S7 and S8.

### 3.3. KEGG Enrichment Analysis for the Proteome

**Figure 3:**
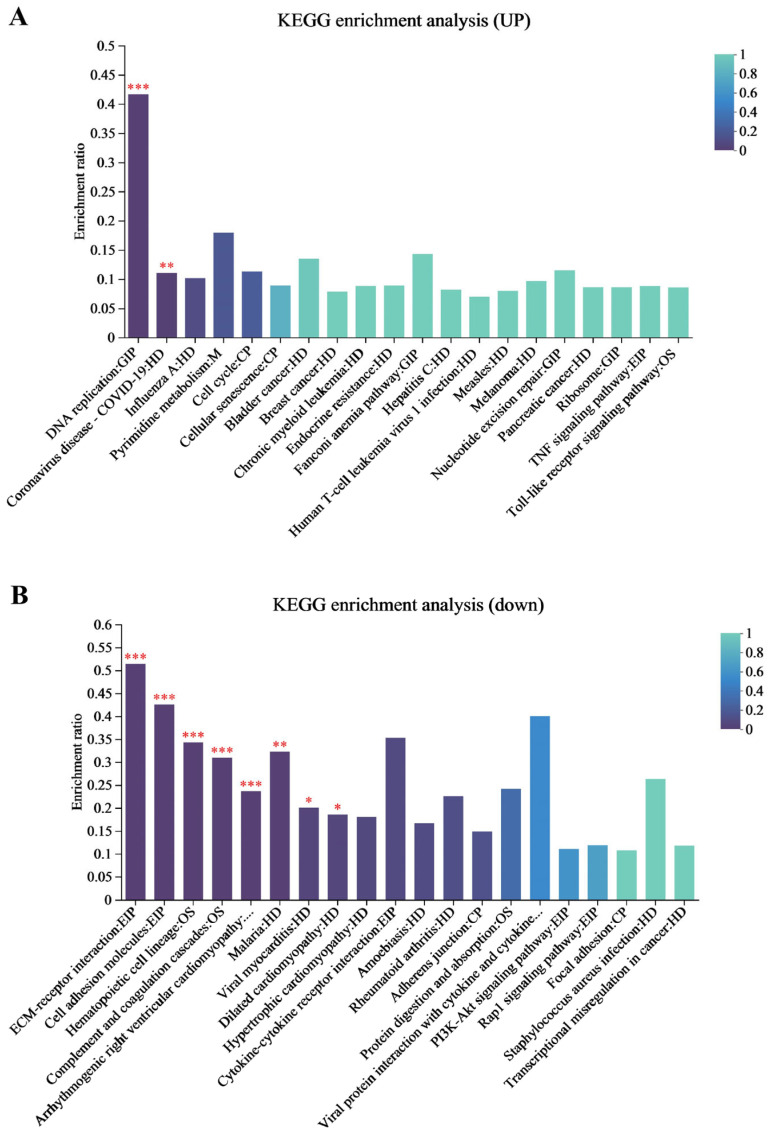
Proteomic KEGG pathways enrichment analysis. (**A**) Proteomic upregulation KEGG enrichment analysis of the 24 h and 48 h groups together. (**B**) Proteomic downregulation KEGG enrichment analysis of the 24 h and 48 h groups together. Note: for the comparison of different groups, *p*-values are those derived from the *t*-test, and “*” indicates significant differences (*p* < 0.05), “**” indicates significant differences (*p* < 0.01), “***” indicates highly significant (*p* < 0.001).

The KEGG pathway enrichment analysis was performed on the DEPs shared between the two infection groups to identify their associated biological functions involved in metabolic and immune pathways during EHV-8 infection. The results of this analysis are presented in Figure 3A, which illustrates the pathway enrichment for the proteome. As shown in Figure 3B, the top 20 most significantly enriched signaling pathways included DNA replication, the TNF signaling pathway, the NF-κB signaling pathway, cellular senescence, endocrine resistance, chronic myeloid leukemia, and T-cell leukemia virus type 1 infection. The DEPs and their related pathways are provided in Supplementary Tables S9 and S10.

### 3.4. Transcriptome and Proteome Association Analysis

**Figure 4:**
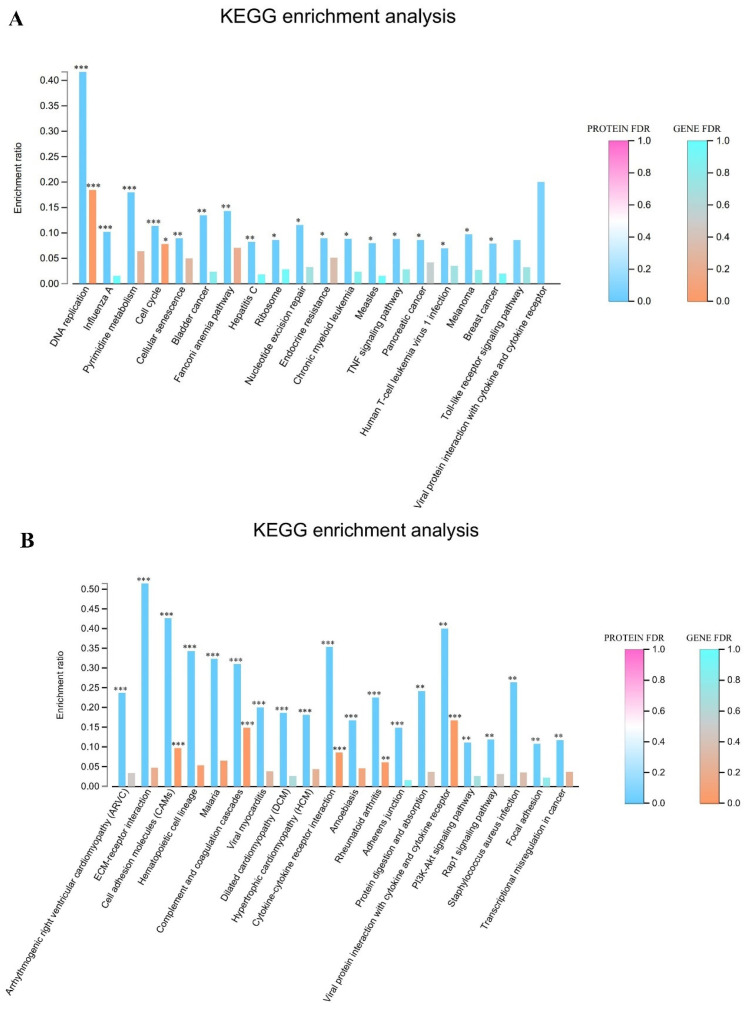
Joint transcriptome and proteome KEGG pathway enrichment analysis map. (**A**) Co-upregulation KEGG enrichment analysis plots for 24 h and 48 h groups. (**B**) Co-downregulation KEGG enrichment analysis in the 24 h and 48 h groups. Note: for the comparison of different groups, *p*-values are those derived from the *t*-test, and “*” indicates significant differences (*p* < 0.05), “**” indicates significant differences (*p* < 0.01), “***” indicates significant differences (*p* < 0.001).

The comprehensive integration of transcriptomic and proteomic datasets was performed to identify coordinately regulated genes and proteins exhibiting concurrent upregulation across both infection groups. The results of this multi-omics comparative analysis are visualized in Figure 4A,B. Pathway enrichment analysis of co-upregulated molecular signatures revealed the significant overrepresentation of several critical biological processes. Prominently enriched pathways included the TNF signaling cascade, which plays a central role in inflammatory response and cell fate determination. DNA replication machinery components showed substantial concurrent elevation at both transcript and protein levels, suggesting enhanced cellular proliferation. Pyrimidine metabolism pathway constituents were similarly co-upregulated, indicating increased nucleotide biosynthesis to support accelerated replication processes. Additionally, significant enrichment was observed in pathways mediating viral protein interactions with cytokines and cytokine receptors, highlighting potential mechanisms of host immune modulation. Multiple additional interconnected biological processes demonstrated coordinated upregulation across both molecular levels, reinforcing the biological significance of these findings and suggesting a coherent cellular response to infection.

### 3.5. Validation of Inflammation Induced by EHV-8 Infection Through the TNF Signaling Pathway

**Figure 5:**
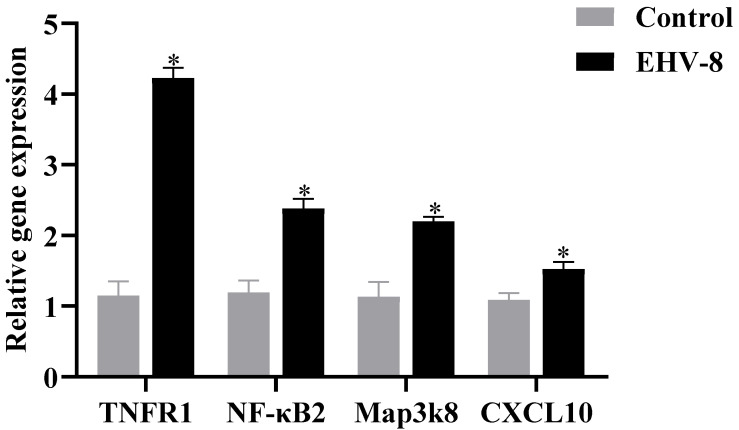
RT-PCR results for 48 h infected groups. Note: for the comparison of different groups, *p*-values are those derived from the *t*-test, and “*” indicates significant differences (*p* < 0.05).

**Figure 6:**
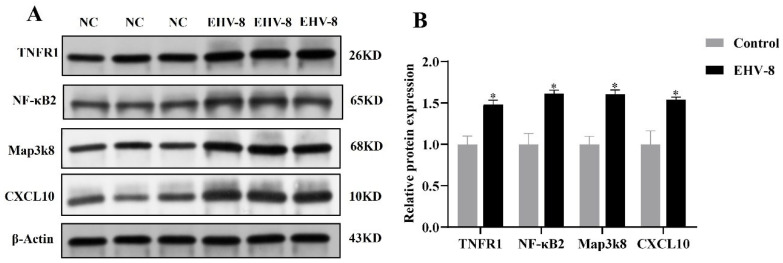
Graph of Western blot results of differential proteins in 48 h infection group. (**A**) WB results. (**B**) Relative expression map of differential proteins. Note: for the comparison of different groups, *p*-values are those derived from the *t*-test, and “*” indicates significant differences (*p* < 0.05).

Transcriptome and proteome association analyses indicated a strong correlation between the TNF signaling pathway and the inflammatory response during the EHV-8 infection of RK-13 cells. To further validate this hypothesis, key components of the TNF signaling pathway, specifically TNFR1, NF-κB2, MAP3K8, and CXCL10, were selected for analysis. These 48 h infectome factors were evaluated at both the transcriptional and protein levels using RT-PCR and PRM, respectively. RT-PCR analysis (Figure 5) revealed that, compared to the control group, the expression levels of TNFR1, NF-κB2, MAP3K8, and CXCL10 were significantly upregulated in the infected group (*p* < 0.05). These results suggest that EHV-8 infection may induce an inflammatory response through the modulation of the TNF signaling pathway. In addition, PRM-based quantitative proteomic analysis, as shown in Table 1, exhibited a similar trend to that observed in the TMT-based quantification, with significant differences detected (*p* < 0.05, FC > 2). As shown in Figure 6, the differential proteins TNFR1, NF-κB2, MAP3K8, and CXCL10 were significantly elevated in the infected group compared to the control group. These findings further corroborate the involvement of the TNF signaling pathway in the inflammatory response induced by EHV-8 infection at the protein level.

## 4. Discussion

Transcriptomics and proteomics analyses provide valuable insights into the overall expression of genes and proteins within cells, offering a comprehensive understanding of cellular responses to various stimuli. Transcriptomic analysis enables the simultaneous detection and quantification of all expressed mRNA transcripts in an organism at a specific point in time, which helps identify the genes associated with particular traits. On the other hand, proteomic analysis provides a direct representation of the proteome and thus reflects the functional translation of genomic information. This approach is particularly useful in identifying the molecular mechanisms involved in disease processes, such as viral infections, and can shed light on host immune responses and regulatory pathways activated during infection [11,12,13].

In this study, we investigated the molecular mechanisms through which EHV-8 infects RK-13 cells by integrating transcriptomic and proteomic data. Our results, obtained from KEGG enrichment analysis, revealed that genes that were significantly upregulated following EHV-8 infection were notably enriched in the TNF signaling pathway. This suggests that the TNF signaling axis plays a central role in mediating the host’s response to EHV-8 infection. TNF is a critical cytokine produced primarily by macrophages and plays key roles in regulating both innate and adaptive immunity, as well as in initiating and resolving of inflammation [14]. It is rapidly released in response to various types of injury and immune stimulation. Functionally, TNF can activate a cascade of inflammatory responses, including the production of other cytokines and chemokines [15].

One of the major transcription factors involved in the immune response is nuclear factor kappa-light-chain-enhancer of activated B cells (NF-κB), which regulates the expression of genes involved in inflammation and immune responses [16]. The activation of NF-κB is essential for the expression of pro-inflammatory cytokines like TNF-α (Tumor necrosis factor alpha), IL-1 (Interleukin-1), and IL-6, as well as chemokines and adhesion molecules such as vascular cell adhesion molecule-1 (VCAM-1), which regulate immune cell recruitment to sites of infection [17]. Previous studies have demonstrated that TNF-α can stimulate endothelial cells to express adhesion molecules like VCAM-1 and ICAM-1 (Intercellular adhesion molecule-1), which are key to promoting inflammation during infection [18]. TNFR1, a primary receptor for TNF-α, activates NF-κB signaling upon binding, which then triggers a variety of biological responses, including inflammation and apoptosis [19]. Our study found that EHV-8 infection significantly upregulated the expression of TNFR1 and NF-κB in RK-13 cells. These findings were validated by RT-PCR and PRM analyses, which confirmed the upregulation of TNFR1 and NF-κB expression in infected cells. This suggests that EHV-8 may enhance the activity of the TNF signaling pathway, thereby promoting a pro-inflammatory response.

In addition to TNF and NF-κB, we also investigated the role of MAP3K8, also known as Cot or Tumor Progression Locus 2 (TLP2) [20]. MAP3K8 is a key regulator of inflammatory responses, with both pro-inflammatory and anti-inflammatory effects, depending on the cellular context. Specifically, MAP3K8 can activate the MEK1/2-ERK1/2 pathway, which is involved in modulating various inflammatory responses. Our results demonstrated that EHV-8 infection significantly upregulated MAP3K8 expression in RK-13 cells, indicating that the virus may contribute to inflammation through the MAP3K8-mediated pathway. Conjoint analysis of the transcriptomic and proteomic data further confirmed that EHV-8 infection induces significant changes in the expression of key proteins within the TNF signaling pathway, including TNFR1, NF-κB, and MAP3K8. This suggests that EHV-8 infection in RK-13 cells is closely linked to the activation of the TNF signaling pathway, which likely plays a central role in the inflammatory response to the virus. This study represents the first comprehensive investigation of the molecular mechanisms underlying EHV-8 infection at both the transcriptomic and proteomic levels. However, it is important to note that the results presented here are based on in vitro data from RK-13 cells and may not fully represent the mechanisms of infection in donkeys, the natural host of EHV-8. Therefore, further in vivo studies in donkeys are needed to validate and extend the findings of this study.

## 5. Conclusions

Altogether, this study provides valuable insights into the molecular mechanisms underlying EHV-8 infection in RK-13 cells, highlighting significant changes in gene and protein expression. Transcriptomic and proteomic analyses revealed extensive alterations in immune and metabolic pathways, particularly in the TNF and NF-κB signaling pathways, which play crucial roles in inflammatory response. Our findings underscore the potential for targeting these pathways when developing therapeutic strategies against EHV-8 and related viral infections.

## Figures and Tables

**Table 1:** PRM quantitative results.

Protein ID	Protein Description	Control	Infection	FC Control vs. Infection	*p*-Value
		RPM Quantification	RPM Quantification		TMT/RPM
ocu04668	TNFR1	0.41 ± 0.12	11.48 ± 2.81	28.05	0.001
ocu04064	NF-κB2	0.43 ± 0.13	9.03 ± 2.75	21.12	0.001
ocu04010	MAP3K8	1.05 ± 0.02	11.06 ± 0.06	10.49	0.034
ocu04010	CXCL10	1.98 ± 0.14	3.79 ± 0.45	7.54	0.043

Note: compared with the control group, the *p*-value is the value obtained from *t*-test, *p* < 0.05, FC < 2.

## Data Availability

All the data we used and analyzed in the current study are available in the manuscript and Supplementary Files. The RNA sequencing data were submitted to NCBI, with the SRA accession number PRJNA1246761. In addition, the proteomics data are available via ProteomeXchange, with the identifier PXD062648.

## Supplementary Materials

The following supporting information can be downloaded at: https://www.mdpi.com/article/10.3390/v17050647/s1. Annex S1: Primer and Antibody Information; Table S1: R2vsR1; Table S2: R3vsR1; Table S3: Transcriptome Common Rise and Fall Data Sheet; Table S4: D2vsD1; Table S5: D3vsD1; Table S6: Proteome Common Rise and Fall Data Sheet; Table S7: Statistical table of KEGG enrichment analysis of down transcriptome; Table S8: Statistical table of KEGG enrichment analysis of up transcriptome; Table S9: Statistical table for KEGG enrichment analysis of the down proteome; Table S10: Statistical table of KEGG enrichment analysis of up proteome.
