# Supplementary material for: Transcriptomic and Proteomic Profiling of Rabbit Kidney Cells Infected with Equine Herpesvirus 8
Source: Viruses. 2025 Apr 29;17(5):647. doi: 10.3390/v17050647 (PMC12115596; doi:10.3390/v17050647)
Supplement: Supplementary file 1 [file viruses-17-00647-s001.zip › viruses-3494223-supplementary/Supplementary File S1/Annex 1-Primer and Antibody Information.docx]

Table 1 Primer sequence information for RT-PCR

| Genes | Sequence**（**5'-3'） |
| --- | --- |
| TNFR1-F  TNFR1-R | GCATGAAGCTCACGGACAAC  GTGAGTGAGGAGCACGTAGG |
| NF-κB2-F  NF-κB2-R | ACATCCATGCGGAGAACGAG  TCACCTTAGTGCTGCGAGTG |
| Map3k8-F  Map3k8-R | AGGAAGCACCGAGGAATCTGAG  CAAGATTGAAGTAGCCAGCCAGAG |
| Cxcl10-F  Cxcl10-R | GCTGCTGCTTCTGCTCCTG  GCCTTCACATTCACACTTTGGATG |
| GAPDH-F  GAPDH-R | AACGGGAAACTCACTGGCAT  TTGAAGTCGCAGGAGACGAC |

Table 2 Western blot Antibody Information

| Antibody name | Diluting factor | Source | Size (kDa) | Brand |
| --- | --- | --- | --- | --- |
| TNFR1 | 1：1000 | rabbit | 26 | Wanleibio |
| NF-κB2 | 1：3000 | rabbit | 65 | Abcam |
| Map3k8 | 1：1000 | rabbit | 68 | GeneTex |
| Cxcl10 | 1：1000 | rabbit | 10 | Abmart |
| β-action | 1：1000 | rabbit | 42 | Wanleibio |
