# Supplementary figures and images for: Transcriptomic and Proteomic Profiling of Rabbit Kidney Cells Infected with Equine Herpesvirus 8
Source: Viruses. 2025 Apr 29;17(5):647. doi: 10.3390/v17050647 (PMC12115596; doi:10.3390/v17050647)

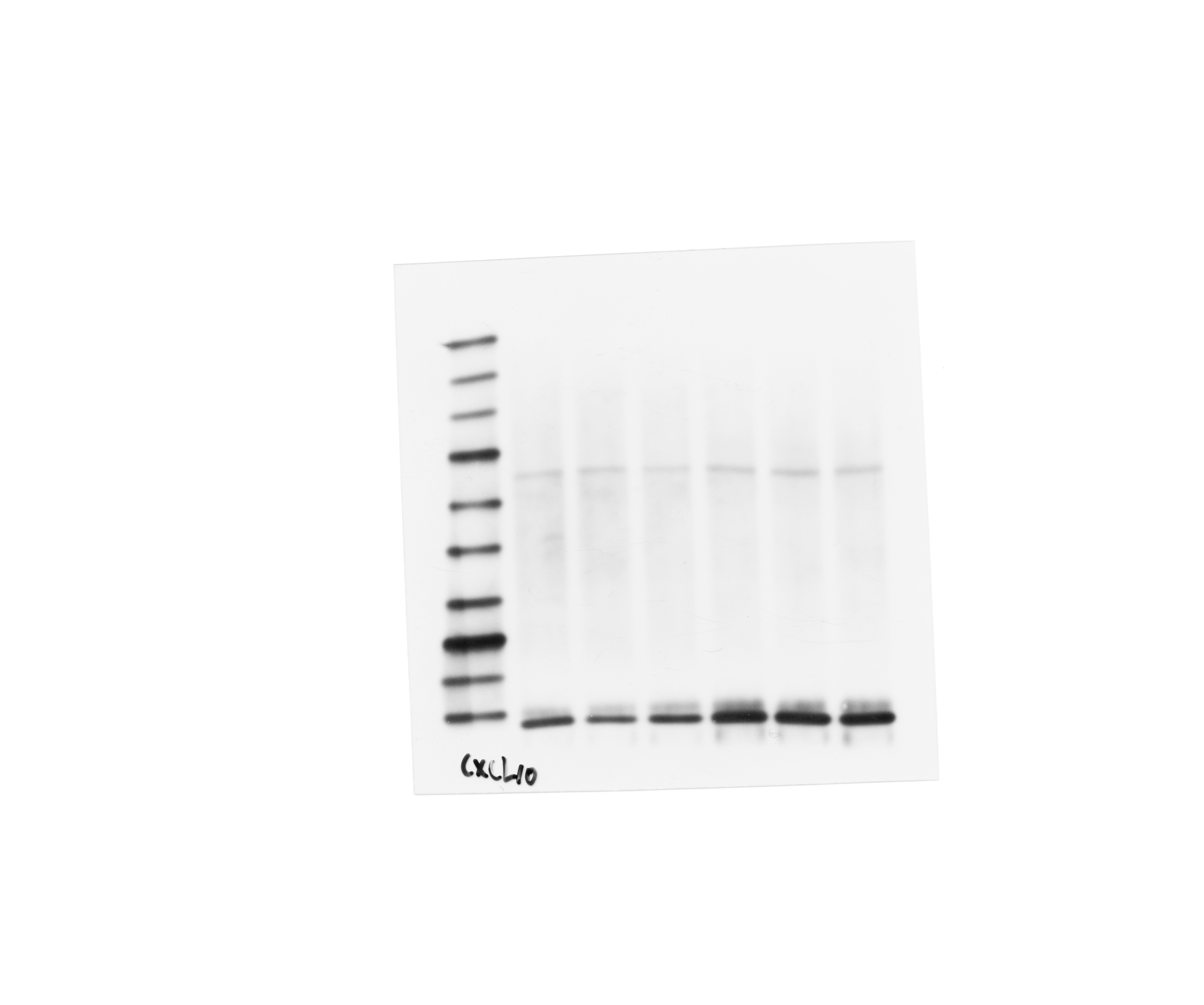

Supplement: Supplementary file 1 [file viruses-17-00647-s001.zip › viruses-3494223-supplementary/Supplementary File S1/WB/CXCL10.tif]

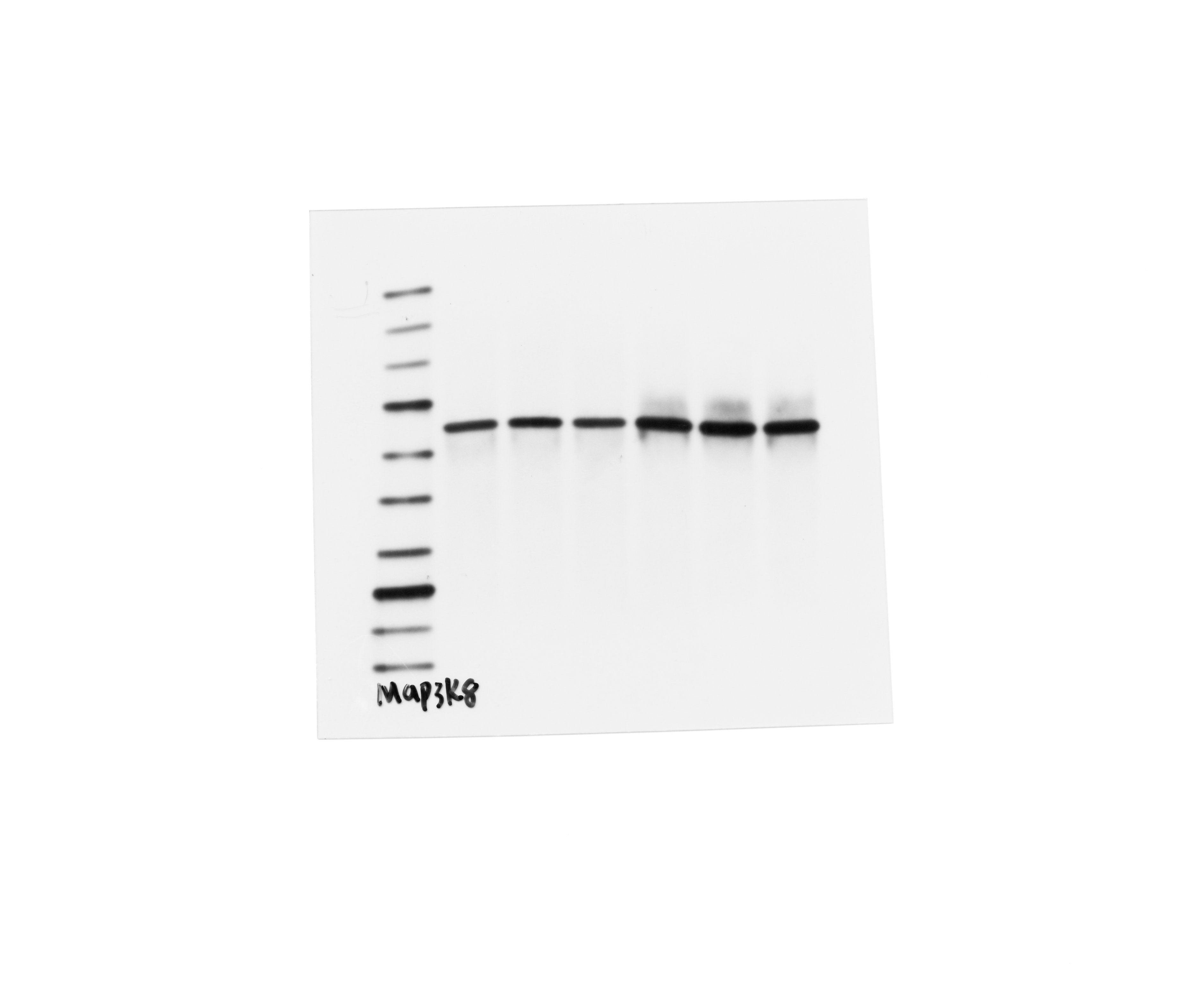

Supplement: Supplementary file 1 [file viruses-17-00647-s001.zip › viruses-3494223-supplementary/Supplementary File S1/WB/Map3k8.tif]

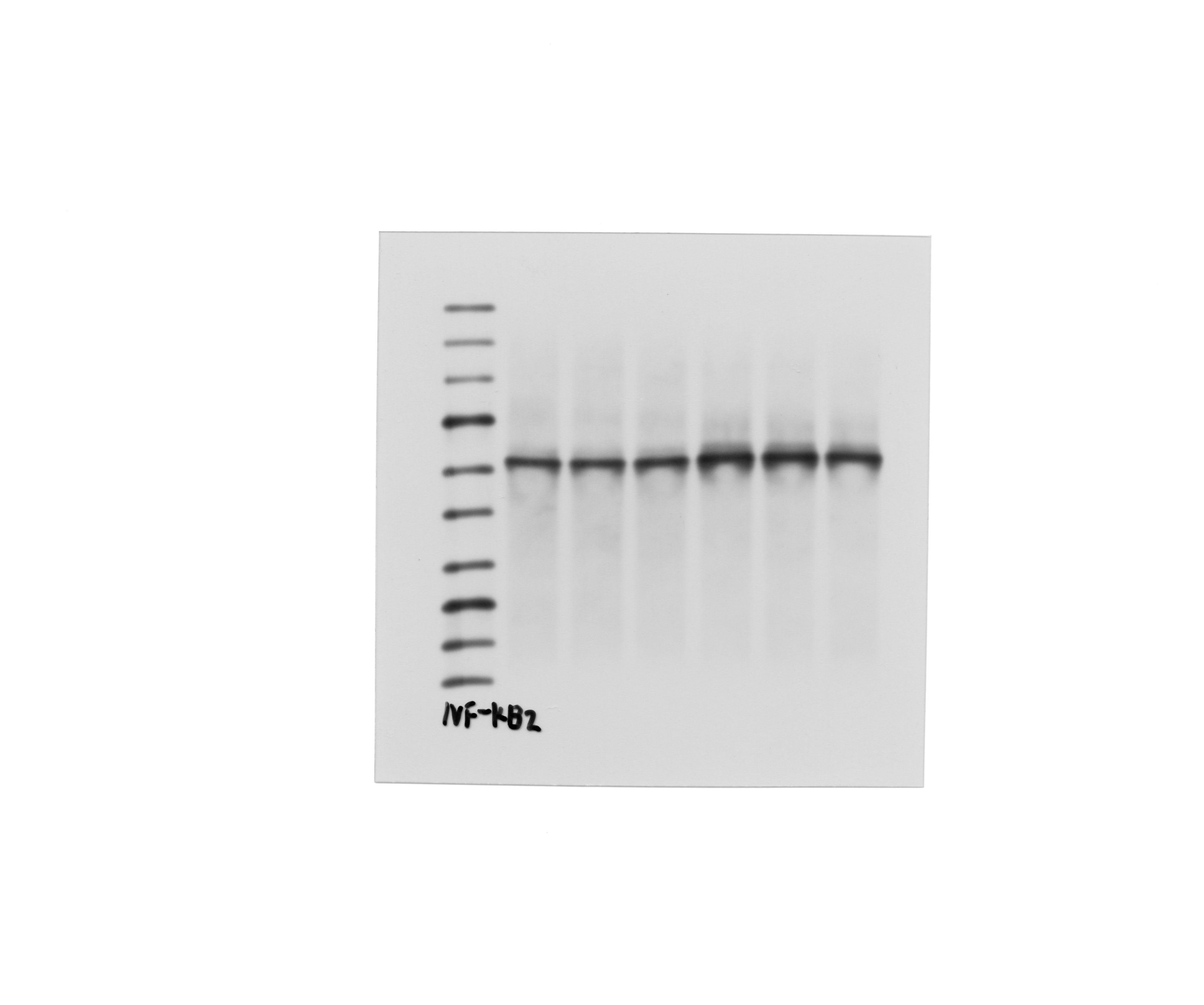

Supplement: Supplementary file 1 [file viruses-17-00647-s001.zip › viruses-3494223-supplementary/Supplementary File S1/WB/NF-κB2.tif]

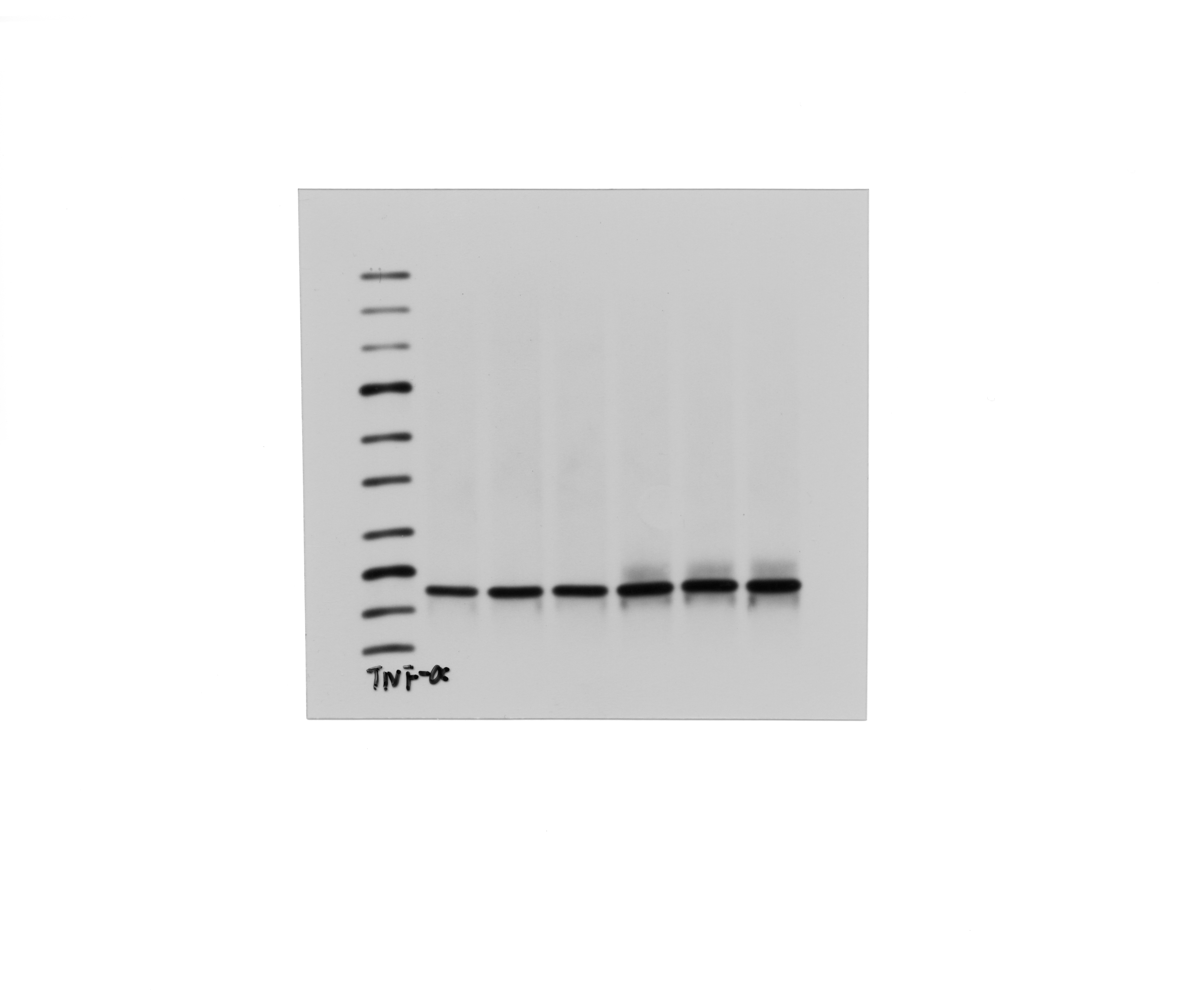

Supplement: Supplementary file 1 [file viruses-17-00647-s001.zip › viruses-3494223-supplementary/Supplementary File S1/WB/TNF-α.tif]

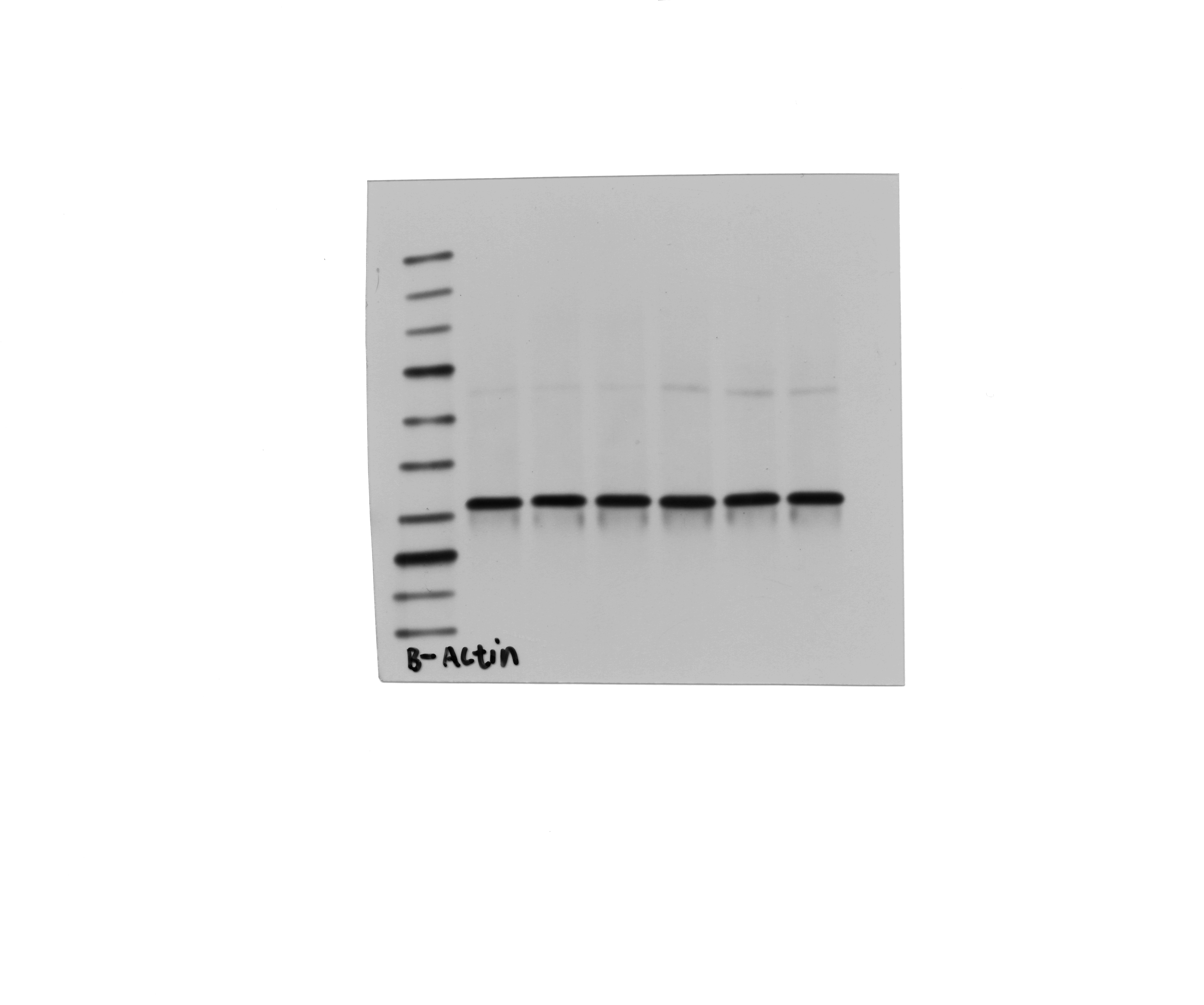

Supplement: Supplementary file 1 [file viruses-17-00647-s001.zip › viruses-3494223-supplementary/Supplementary File S1/WB/β-Actin.tif]
